# A commentary on Zuniga‐Montanez and Davies et al.: how did COVID‐19 affect young children's language environment and language development? A scoping review

**DOI:** 10.1111/jcpp.14132

**Published:** 2025-02-22

**Authors:** Hamish Chalmers

**Affiliations:** ^1^ Department of Education University of Oxford Oxford UK

## Abstract

It was early 2020, a week or two into Hilary Term, what everyone else calls Spring Term, but we at Oxford love our arcane traditions. I recall one of my graduate students, from China, coming to me ashen‐faced at the end one of my lectures on the effects of bilingualism on the linguistic and cognitive development of young learners. “Please be careful,” she said. “Have you heard about the disease. It's really scary. Please look after your family.” Over the preceding Christmas break, news had started to filter through about a new form of flu that had spread rapidly from Wuhan in Eastern China to other parts of the country and was now starting to emerge in other parts of the world. We were starting to see desperate images of enforced quarantine, coerced separation of infected individuals from their loved ones, the rapid construction of temporary hospitals to house the unwell, and of course, school closures. It didn't look good. But I had seen similar outbreaks in the past. I had been working in Southeast Asia during the avian flu epidemic of 2003–04, and I was still there when swine flu broke out in 2009. Both were worrying, but neither had come to anything that could be classified as universally threatening. The school where I worked sent colleagues and children to be tested at the first sign of a tickly throat or stuffy nose, and a strict and regular cleaning and hand sanitising regime was implemented.

It was early 2020, a week or two into Hilary Term, what everyone else calls Spring Term, but we at Oxford love our arcane traditions. I recall one of my graduate students, from China, coming to me ashen‐faced at the end one of my lectures on the effects of bilingualism on the linguistic and cognitive development of young learners. “Please be careful,” she said. “Have you heard about the disease. It's really scary. Please look after your family.” Over the preceding Christmas break, news had started to filter through about a new form of flu that had spread rapidly from Wuhan in Eastern China to other parts of the country and was now starting to emerge in other parts of the world. We were starting to see desperate images of enforced quarantine, coerced separation of infected individuals from their loved ones, the rapid construction of temporary hospitals to house the unwell, and of course, school closures. It didn't look good. But I had seen similar outbreaks in the past. I had been working in Southeast Asia during the avian flu epidemic of 2003–04, and I was still there when swine flu broke out in 2009. Both were worrying, but neither had come to anything that could be classified as universally threatening. The school where I worked sent colleagues and children to be tested at the first sign of a tickly throat or stuffy nose, and a strict and regular cleaning and hand sanitising regime was implemented. No one I knew, or knew of, ever succumbed. The mild panic subsided almost as quickly as it had arisen. Arrogantly, perhaps, but not without some sense of evidence (albeit personal anecdote and experience), I reassured my student that I thought everything would be fine. We had a government that would ensure that effective steps would be taken to minimise if not contain the threat to us in the UK. I assumed that, like those other two epidemics, this one would be over before we knew it. How wrong I was … on both counts.

As everyone now knows, the global COVID‐19 pandemic, officially classified as such by the World Health Organization on 11 March 2020 (Cucinotta & Vanelli, 2020), was not a flash in the pan. It was not an obscure disease that would have a limited impact in a limited number of jurisdictions. It certainly would not stop at the doors of western, educated, industrialised, rich and democratic (WEIRD) nations, as much of the discourse and implied exceptionalism (like my own regrettable assumptions) supposed it would. Moreover, the effects of COVID‐19 would not be limited merely to the biomedical.

The proximal effect of contracting the disease was, of course, on the physical health of the individuals who had it. When the Worldometer coronavirus tracker (https://www.worldometers.info/coronavirus/) stopped counting in April 2024, in excess of 7 million people had died of the disease. Countless more suffered less dramatic effects, and many others continue to suffer at the hands of long COVID‐19. But the effects of COVID‐19, and importantly the world's governments' responses to it, were much further reaching and affected much more than just our physical health.

In their report into the societal effects of the pandemic for the UK's Government Office for Science, the British Academy (2021) identified nine key areas where responses to the pandemic would likely have long‐term impacts. Among these were an exacerbation of existing structural inequalities, such as income and poverty, social deprivation and education. To this latter aspect, they stated:

The consequences of lost access to education at all levels, coupled with changes to assessments, will be felt for years to come, and wholly recovering lost education is unfeasible. (British Academy, 2021, p. 9)

Digging deeper into the effects of lost educational opportunities, they suggested that the effects of closing education institutions, such as schools and Early Years providers, and delivering compulsory education in the home through digital platforms “has been to exacerbate existing socioeconomic inequalities in attainment” (ibid. p. 135) and that that which has been lost will be evident in GCSE and post‐16 qualifications over the next decade.

These estimates of the potential effects on education were made in March of 2021, only a year after the pandemic began. At that point they can only have been predictions. In the years since, we have seen (both anecdotally and empirically) these predictions come to pass. Talking to the BBC in 2024, an Early Years teacher described children arriving at school lacking the expected social and linguistic skills “to communicate with others, with developing their friendships, with their resilience when things are tricky, and talking as well.” (BBC, 2024, n.p.). Large scale studies have supported observations of this sort. Investigations into school readiness of pandemic era children estimate a 13 percentage point difference in the number of children ‘school ready’ on entering formal education when comparing pre‐ and post‐pandemic rates, a difference that grows to 16 percentage points for children from language minority backgrounds (Powell et al., 2024; Tracey et al., 2022). A review of the relevant evidence on the effects of school closures in England (Major, Eyles, Lillywhite, & Machin, 2024) predicts “the biggest overall decline in basic GCSE achievement for at least 2 decades” (p. 4). Effects that will be felt “well into the 2030s” (ibid.).

It is only 5 years since the pandemic started. Our understanding of its long‐term impacts, therefore, is inevitably somewhat speculative. But as each day passes, new evidence comes to light that will allow us to develop an ever more nuanced understanding of where pandemic‐related problems lie and, therefore, where we might best expend our energies on trying to ameliorate them. Despite the British Academy's early warning that educational losses are likely to be irrevocable, there is nonetheless a duty on the part of the scientific community to try to understand what happened during the pandemic, to look carefully at where those losses, if present, are evident, and to set research and policy agendas that aim to address them. It is this critical gap in our understanding where systematic reviews like Zuniga‐Montanez and Davies et al.'s review into the effects of COVID‐19 on language environment and language development play a crucial role.

Zuniga‐Montanez and Davies et al.'s review makes an important contribution to our understanding of the field. This contribution is not just because it addresses an important topic with far‐reaching implications for the educational journeys of young people all over the world, but because its methodological approach and clarity of reporting engender trust in the process and, therefore, its findings. It also provides an excellent model for others wishing to make robust contributions to the state of our knowledge on this topic and related ones.

Systematic review is an umbrella term that refers to “a way of bringing together what is known from the research literature using explicit and accountable methods” (Gough, Thomas, & Oliver, 2012, p. 1). Broadly speaking, systematic reviews “aim to find as much as possible of the research relevant to the particular research questions, and use explicit methods to identify what can reliably be said on the basis of these studies” (EPPI Centre, 2016, n.p.). Applied linguistics, one of the fields in which Zuniga‐Montanez and Davies et al.'s review sits, has seen rapid growth of this type of research since the turn of the millennium (Chalmers, Brown, & Koryakina, 2024). Under this methodological umbrella, the aims and scopes of systematic reviews have evolved to include reviews that adopt a variety of different approaches to meeting a variety of different aims. In addition to the more traditional ‘what works’ review, we now see systematic reviews that synthesise qualitative evidence, reviews that survey the methodological approaches used in a field of enquiry, gap maps that identify under‐researched areas within a larger topic, realist syntheses that combine quantitative findings with qualitative findings to assess what works, for whom, and under what conditions, and many more (Chong & Plonsky, 2024). What they all share … or should share … is a commitment to using and reporting explicit and accountable methods.

However, this growth in type and topic has not been matched by a growth in rigour; neither in the conduct nor the reporting of these reviews. An audit of the methodological and reporting quality of systematic reviews in language education (Chalmers et al., 2024) found that most systematic reviews in the field failed to include sufficient information to understand how they were conducted. The majority also failed to include aspects of the method that are widely considered to be crucial for meeting its main aim: ‘to identify what can reliably be said’ about the evidence on a given topic of enquiry. Concern about failures to conduct and report systematic reviews completely and transparently is not just a matter of authorial pedantry, but has real world consequences for policy and practice, and therefore for the lives of people who stand to benefit from an accurate understanding of the evidence on which that policy and practice is based. Knowing, with confidence, what can be reliably said about an area of inquiry allows practitioners and policy makers to plan social and educational interventions that address the issues that have emerged from a complete and clear assessment of the evidence. It allows researchers to conduct new primary research that builds profitably on what we already know, address gaps in our understanding, and, ideally, improve the lives of the people who are affected by the decisions it informs. Moreover, in the relatively embryonic field of research into the effects of the COVID‐19 pandemic, clear methodological reporting allows the original authors or a new review team to update the review as new evidence comes to light, as it inevitably will.

The aim of Zuniga‐Montanez and Davies et al.'s well‐conducted and well‐reported review was to provide a preliminary assessment of the size and scope of the available research literature on a new and fast‐moving area of enquiry, and to draw such conclusions that are available on the basis of that emerging evidence. Their primary purpose was not necessarily to provide a definitive answer to the overarching question, but rather to show the extent and nature of research on the effects of the COVID‐19 pandemic as it relates to young children's language environment and language development. Their method thus allows readers to identify areas that have been well‐covered, or alternatively, suggest where more work is needed. The review also identifies where in the world eligible research has been conducted and provides a meta‐view of the methodological approaches taken in the assembled primary research. All of this is informative in its own right, but it also stands as a potential precursor to in‐depth reviews, where sub‐themes from within the larger literature can be analysed in more detail, the methodological quality of the primary research can be assessed through risk of bias appraisals, and fine‐grained statistical or narrative synthesis can be applied to the data, or sub‐sets of it.

In the reporting of their review, Zuniga‐Montanez and Davies et al. have followed a number of best practices in the field. First, they have used the PRISMA (Preferred Reporting Items for Systematic Reviews and Meta‐Analyses) extension for scoping reviews (Trico et al., 2018) to inform the conduct and reporting of their research. The use of PRISMA ensures that reviews are reported such that the method is clear and complete. It also exerts a washback effect on method: that which is reported must first be conducted. This allows confidence on the part of the reader that they understand the terms under which the review was prepared. Moreover, if they find that critique is necessary, this procedure can be done in full possession of the facts of that conduct, rather than having to guess, as was the case in so many of the reviews identified in Chalmers et al.'s (2024) audit.

Second, they prepared and prospectively published a protocol for the review. A protocol helps to ensure that the review team has a common point of reference while conducting the review, which supports both efficiency and effectiveness throughout the process. More importantly, prospective publication of review protocols guards against so‐called “questionable research practices”, such as selective outcome reporting, outcome switching, and publication bias. Thus, the prospective publication of their protocol not only signals Zuniga‐Montanez and Davies et al.'s seriousness about conducting transparent and accountable science, but it allows readers to refer to the protocol to assess whether what was ultimately published faithfully reflects what was planned.

In another demonstration of adherence to the principles of open science, the authors made key materials crucial to understanding their method and findings publicly available on the Open Science Framework (https://osf.io). The Boolean strings used to query the different databases they searched are provided in full, allowing for the search to be checked by peer reviewers and interested readers. It also makes faithful replication or updating by other review teams possible. Again, in the still‐evolving scenario around the emerging effects of COVID‐19 on children's language environment and language development, this method allows us to maintain an up‐to‐date understanding of how the pandemic affected young learners and inform ongoing research, policy and practice decisions accordingly.

There is inevitably limited space that journals can devote to any one article. A topic as large as the effects of the global pandemic on language development cannot be exhausted in one paper. Moreover, the way in which Zuniga‐Montanez and Davies et al. chose to frame the syntheses of the evidence they located will have been informed by what they see as the priorities. Similarly, the conclusions and implications they have reached after interpreting that evidence will have been informed by their own priorities and biases. See, for example, their reference to what they describe as “the word gap *ideology*” (p. 32, emphasis added). This framing is subjective. Interpretation, in this instance, depends on whether one believes that evaluation of the evidence on the nature and extent of early exposure to language is ideological and political or, alternatively, rational and dispassionate. They are entitled to their interpretation, but in another demonstration of best practice, they have made their complete data extraction table freely available so that others can interrogate the evidence to see if they come to the same conclusions.

The dataset is extremely detailed, including information about the individual study aims, geographical locations, sample ages, sample sizes, sample characteristics, data collection periods, study designs, analysis methods, and many more. As well as allowing for alternative interpretations of those data, it allows others to dig into specific areas within the review, and thus to elaborate on just one of the many themes they uncovered. Making these data freely available is not just a matter of professional generosity; it adheres to the principle that knowledge is always provisional and we are constantly refining our understanding of the world. They have dipped the giant's shoulder to make it easier for others to stand on it.

In sum, Zuniga‐Montanez and Davies et al.'s review offers a valuable example of best practices in the conduct and reporting of evidence syntheses that, sadly, are not routine in many social scientific fields. Their review fosters confidence because they have adhered to a standardised method that supports clear and complete reporting. It offers solid foundations on which to build our understanding of the challenges experienced by the young people whose educational lives were disrupted by the COVID‐19 pandemic and societal responses to it. And it provides the starting point for others to see if the British Academy can be proved wrong about the feasibility of ever recovering. I hope others see the value of such scrupulous work and use it as a model for their own.
